# Prostate Cancer Mortality Associated with Aggregate Polymorphisms in Androgen-Regulating Genes: The Atherosclerosis Risk in the Communities (ARIC) Study

**DOI:** 10.3390/cancers13081958

**Published:** 2021-04-19

**Authors:** Anna E. Prizment, Sean McSweeney, Nathan Pankratz, Corinne E. Joshu, Justin H. Hwang, Elizabeth A. Platz, Charles J. Ryan

**Affiliations:** 1 Division of Hematology, Oncology and Transplantation, University of Minnesota Medical School, Minneapolis, MN 55455, USA; jhwang@umn.edu (J.H.H.); ryanc@umn.edu (C.J.R.); 2 University of Minnesota Masonic Cancer Center, Minneapolis, MN 55455, USA; 3 University of Minnesota Medical School, Minneapolis, MN 55455, USA; mcswe027@umn.edu; 4 Department of Laboratory Medicine and Pathology, University of Minnesota, Minneapolis, MN 55455, USA; pankr018@umn.edu; 5 Department of Epidemiology, Johns Hopkins Bloomberg School of Public Health, Baltimore, MD 21205, USA; cjoshu1@jhu.edu (C.E.J.); eplatz1@jhu.edu (E.A.P.); 6 Sidney Kimmel Comprehensive Cancer Center at Johns Hopkins, Baltimore, MD 21287, USA; 7 Department of Urology, The James Buchanan Brady Urological Institute, Johns Hopkins University School of Medicine, Baltimore, MD 21287, USA

**Keywords:** androgen, androgen-regulating genes, genetic polymorphisms, genetic risk score, prostate cancer, survival

## Abstract

**Simple Summary:**

The growth of prostate cancer is driven by androgens through activation of the androgen receptors and its target genes in the prostate tumor tissue. Alterations in several genes involved in androgen regulation may influence the prognosis of prostate cancer, but have not been tested in a population-based study. In this epidemiological study, we confirmed that the alteration in the *HSD3B1* gene that affected the progression of prostate cancer in clinical trials, is associated with increased risk of prostate cancer death in men diagnosed with metastatic disease. Additionally, we examined the cumulative effect of pre-specified alterations in the three critical androgen-regulating genes (including *HSD3B1*) and found that their combination predisposes to death from prostate cancer. Our findings suggest that an interplay between the androgen-regulating genes may influence the prognosis of prostate cancer and should be further examined for tailoring prostate cancer treatment.

**Abstract:**

Genetic variations in androgen metabolism may influence prostate cancer (PC) prognosis. Clinical studies consistently linked PC prognosis with four single nucleotide polymorphisms (SNPs) in the critical androgen-regulating genes: 3-beta-hydroxysteroid dehydrogenase (*HSD3B1*) rs1047303, 5-alpha-reductase 2 (*SRD5A2*) rs523349, and solute carrier organic ion (*SLCO2B1*) rs1789693 and rs12422149. We tested the association of four androgen-regulating SNPs, individually and combined, with PC-specific mortality in the ARIC population-based prospective cohort. Men diagnosed with PC (N = 622; 79% White, 21% Black) were followed for death (N = 350) including PC death (N = 74). Cox proportional hazards regression was used to estimate hazard ratios (HR) and 95%CI adjusting for center, age, stage, and grade at diagnosis using separate hazards for races. A priori genetic risk score (GRS) was created as the unweighted sum of risk alleles in the four pre-selected SNPs. The gain-of-function rs1047303C allele was associated PC-specific mortality among men with metastatic PC at diagnosis (HR = 4.89 per risk allele, *p* = 0.01). Higher GRS was associated with PC-specific mortality (per risk allele: HR = 1.26, *p* = 0.03). We confirmed that the gain-of-function allele in *HSD3B1* rs1047303 is associated with greater PC mortality in men with metastatic disease. Additionally, our findings suggest a cumulative effect of androgen-regulating genes on PC-specific mortality; however, further validation is required.

## 1. Introduction

Growth of prostate cancer (PC) is driven by androgens through activation of the androgen receptor and its target genes. Because progression of PC is dependent on androgen supply, a mainstay therapy for patients with metastatic PC is androgen deprivation therapy (ADT) and the direct targeting of the androgen receptor via pharmacologic antagonists. Additional blockade of androgen production within the tumor may be achieved by abiraterone acetate via inhibiting a steroid enzyme—cytochrome P450 17 alpha-hydroxylase CYP17) [1]. Evaluating associations of variants in genes involved in “androgen supply chain”—androgen production, uptake, and conversion—with outcomes after a PC diagnosis may help to elucidate the potential role, singularly or in aggregate, of these pathways in PC progression.

We hypothesized that four single nucleotide polymorphisms (SNPs) in three critical androgen-regulating genes: 3-beta-hydroxysteroid dehydrogenase (*HSD3B1*), solute carrier organic ion (*SLCO2B1*), and steroid 5-α-reductase type 2 *(SRD5A2*) [2,3,4,5,6], contribute, individually and in aggregate, to PC outcomes. *HSD3B1* encodes an enzyme involved in the de novo synthesis of androgens in PC cells and adrenal gland, and serves as a rate-limiting step in the production of the highly potent androgen receptor agonist—dihydrotestosterone (DHT) in PC tumor [5,7,8]. The *HSD3B1* 1245A→C polymorphism (rs1047303), resulting in a change in amino acid (Asn367Thr), leads to increased DHT production by promoting stability of the enzyme 3α-hydroxysteroid dehydrogenase 1 (3βHSD1) [5,9]. Consistent with this mechanism, 1245A→C polymorphism was associated with a shorter time to prostate-specific antigen (PSA) recurrence, metastatic progression, and reduced overall survival of PC patients treated with ADT [4,5,10,11]. A second androgen-regulating gene, *SLCO2B1*, encodes a member of the organic ion transporter protein (OATP) family, and facilitates cellular uptake of androgens including testosterone and DHT [2]. The levels of *SLCO2B1* expression were shown to correlate with uptake of dehydroepiandrosterone (DHEA) by PC cells, as well as drugs such as abiraterone [12,13]. In the exonic *SLCO2B1* SNP, rs12422149, involving A→G transition (Arg312Gln), the more active GG genotype was associated with PC progression in patients receiving ADT [13,14]. Another *SLCO2B1* SNP, intronic rs1789693, was also associated with progression-free survival [13] and overall survival in PC patients receiving ADT [14], but the findings in those papers were inconsistent. A third gene, *SRD5A2*, codes for 5-alpha-reductase 2 enzyme converting testosterone to DHT [6]. This gene contains an exonic SNP, rs523349 involving G→C transversion (V89L). In a Japanese study of metastatic PC patients treated with ADT, the more active GG genotype was associated with a higher risk of progression of PC and death [6], although not all findings were consistent [15].

In the population-based study—Atherosclerosis Risk in Communities (ARIC), we focused on four pre-specified SNPs in the three critical androgen-regulatory genes, based on their biological plausibility and their clinical importance, because all of them showed associations with PC progression and mortality among men treated with androgen inhibitors. We confirmed that the gain-of-function allele in *HSD3B1* rs1047303 is associated with greater PC mortality in men with metastatic disease. This confirmation in population-based study is important because this study included more diverse participants with longer follow-up as compared to PC patients typically enrolled in therapeutic trials. In addition, we conducted an exploratory analysis of three other pre-specified androgen-regulating SNPs in relation to PC-specific and all-cause mortality, individually and in combination. We hypothesized that the genetic variants involved in androgen production, uptake and conversion, would have a cumulative effect on the PC-specific mortality. We found that the genetic risk score combined of androgen-regulating SNPs (including *HSD3B1* rs1047303) is associated with increased PC-specific mortality in individuals with PC irrespective of their age and stage at diagnosis. These findings suggest the potential importance of these polymorphisms for treatment guidelines, requiring future validation in large studies.

## 2. Materials and Methods

### 2.1. ARIC Study Design

ARIC is a prospective cohort that enrolled 15,792 participants 45 to 64 years old from four U.S. communities in 1987-89 with a goal to examine the causes and effects of atherosclerosis [16,17]. Study participants were recruited from Forsyth County, NC; Jackson, MS; Washington County, MD; and the suburban areas of Minneapolis, MN. Within each community, approximately 4000 participants were selected by probability sampling. Unlike other communities, only blacks were sampled in Jackson and oversampled in Forsyth County, so that the race-specific estimates may be derived. The participants were followed longitudinally with frequent examinations that collected demographic, lifestyle and clinical data. Response rates were 90–99% for the annual follow-up calls and 83–90% for semi-annual follow-up calls among living participants who have not withdrawn consent to be contacted [18]. PC cases were ascertained from 1987–2015 by linkage with state cancer registries and supplemented by active cohort follow-up [19]. All sources of data were adjudicated using standardized protocols [19]. Deaths, including from PC as the underlying cause, were obtained from the National Death Index through 31 December 2019. Written informed consent was obtained from each participant, and all participating institutional review boards approved the study protocol.

In the ARIC study, of 7082 men, 369 men had prevalent cancer at the study baseline and were excluded from this analysis. Of remaining 6713 men, 952 developed PC during follow-up. Those without information on genotyping (N = 176) and the stage of diagnosis (N = 154) were excluded, yielding 622 men with PC. About 97% PC cases with missing stage were diagnosed before 2006. This is because in earlier years of follow-up, not all ARIC centers even have cancer registries (such as Jackson, Mississippi) and T1c PC cases (detected by PSA elevation only) were coded as unstaged if they were not fully worked up for metastases, as recommended by North American Association of Central Cancer Registries. Among 622 men with PC, 398 (64%) had information about first course of treatment (radiation, surgery and hormone therapy) available from cancer registries.

### 2.2. SNP Genotyping

SNPs were genotyped using the Affymetrix Genome-Wide Human SNP Array 6.0 as discussed in detail previously [20]. Samples were excluded for an insufficient call rate, sex mismatches, discordance with previously genotyped markers, first-degree relatives of an included individual, and genetic outliers. To increase the number of SNPs beyond those on the genotype array, ARIC conducted race-specific imputation of variant dosages to the 1000 Genomes Project Phase I version 3 reference panel [21,22]. Principal components based on the GWAS data were generated by EIGENSTRAT7 to reflect the population structure or genetic ancestry of the ARIC participants [21]. The quality of imputation was high (Rsq > 0.98) in both White and Black men for three of the variants (rs1047303, rs523349, and rs12422149). Imputation accuracy was lower but still acceptable for rs1789693 (Rsq = 0.72 in Whites and 0.78 in Blacks).

### 2.3. Statistical Analysis

We examined associations of each SNP (rs1047303, rs523349, rs1789693, and rs12422149) individually and in aggregate with PC-specific and all-cause mortality in men with a pathologically confirmed PC diagnosis. For each SNP, Kaplan–Meier analysis was conducted using product limit estimate. The follow-up for death has been complete in ARIC, as all deaths have been adjudicated via National death index. Kaplan–Meier were compared between genotypes using the log-rank test. To preserve power in Kaplan–Meier analysis, SNPs were categorized into (1) homozygous for the minor allele combined with heterozygous (i.e., carriers of the minor allele), and (2) homozygous for the major allele (i.e., non-carriers of the minor allele). Since rs1789693 was fully imputed (continuous variable, range 0–2), it was categorized into three groups [minor allele TT (0–0.49), TA (0.5–1.49), and major AA (1.5–2)], and then TA and TT genotypes were combined and compared to the AA genotype. Our primary objective was to confirm the association between exonic rs1047303 and PC-specific mortality in population-based study. Because rs1047303C allele was previously shown to affect PC progression among metastatic cancer patients [5,10,11], we reran the analysis for rs1047303 by limiting to metastatic stage (stage 4) at diagnosis.

Cox proportional hazards regression was used to estimate the hazard ratio (HR) and 95% confidence interval (CI) for PC-specific and all-cause mortality using separate hazards for races. In the main analysis, each SNP was modeled as continuous (range 0–2) assuming an additive genetic model. In addition, to make this analysis consistent with the Kaplan–Meier analysis, each SNPs was dichotomized as in the Kaplan–Meier analysis. Follow-up started at the date of diagnosis or blood collection (22 men (3.7%) had blood collected after diagnosis) and ended at the date of death or the end of 2018, whichever occurred first. Of note, time of blood collection was used as a starting point if blood collection occurred after cancer diagnosis in order not to introduce bias that could arise due to the death between diagnosis and blood collection. For PC-specific mortality, men were also censored at the date of death due to other causes. Proportional hazards assumption was tested for each model using an interaction term between each SNP and follow-up time up on a linear scale; *p*-value < 0.05 was considered as a violation of the assumption. The assumption was not violated for any SNP.

All SNPs appeared to be functional: three SNPs were exonic, and the fourth—intronic SNP rs1789693—was associated with drug tissue levels and minimal residual disease in in vitro study of PC patients receiving abiraterone [12]. Thus, we combined White and Black men and modeled race-specific hazard functions (using “Strata” statement in SAS). We did not adjust for ancestry biomarkers in the main analysis because they were not associated with the outcome or the 4 SNPs, and adjustment for genetic ancestry did not change the inferences. In a sensitivity analysis, we stratified the models by race and then meta-analyzed the regression estimates, with similar inferences to the approach of modeling race-specific hazard ratios. Hence, we present the latter approach here, which was more efficient. Further, we conducted a competing risk analysis for PC–specific mortality using Fine and Gray method because death from other causes may be a competing event [23]. The estimates and *p*-values were similar to those in the analysis that did not account for competing risk, so the latter findings have been presented.

The analyses were adjusted for age (continuous), TNM stage (categories: 1 + 2 combined, 3 and 4) and SEER grade at diagnosis. SEER grade includes the following categories: 1 (corresponds to Gleason sum ≤ 3), 2 (Gleason sum of 4–6), 3 (Gleason grade ≥ 7), We ran two analyses by excluding 35 participants (5.6%) with missing grade and by presenting missing grade as a separate category. The interference was very similar, and, therefore, to preserve power, the analysis with missing grade as a separate category has been presented.

All the categories in the analysis were determined a priori. Although confounding is not expected in gene-outcome associations, we tested whether additional adjustment for BMI, smoking, history of diabetes and cardiovascular diseases, and first course treatment (surgery, radiation and hormonal treatment) changed the associations. Because all HRs did not markedly change (<10%) after additional adjustment, those variables were not included in the final models.

All the analyses were conducted using SAS version 9.4 (SAS Institute, Inc., Cary, NC, USA), significant two-sided *p*-values were set at <0.05. No correction for multiple comparisons was made given that each SNP was pre-selected based on its link with androgen metabolism and prostate cancer, and our main objectives were to (1) confirm the effect of *HSD3B1* rs1047303 and (2) examine the cumulative effect of those SNPs (combined into the genetic risk score, GRS) rather than the effect of each individual SNP.

#### 2.3.1. Genetic Risk Score (GRS)

To evaluate the potential cumulative effect of the SNPs, we created a priori GRS by summing all four SNPs under study. Because all SNPs’ values were genotyped or imputed, there have been no missing values for any SNP under study. An allele was considered a risk allele if it was involved in androgen uptake, conversion and production or was more actively involved in the metabolism of hormone therapy for PC [12]. We calculated the HRs for PC-specific and all-cause mortality associated with the GRS analyzed as continuous and as a categorical variable (tertiles). In addition to examining survival over the full follow-up, we estimated the 10-year survival due to its importance in men with PC. Finally, we examined whether including the GRS in the multivariable model improved the model fit by testing differences in the likelihood ratios between the models.

#### 2.3.2. Sensitivity Analyses

We conducted additional sensitivity analyses. First, we ran the analyses separately in Whites and Blacks. However, the number of outcomes in Black men was limited (16 deaths from PC of 61 total deaths), precluding stable estimates for some analyses. Second, we re-ran the analyses using the follow-up time ending in 2012 (instead of 2019) to account for confounding that could have been introduced by the approval of abiraterone as a therapeutic for reducing adrenal androgen production, during this time [1,24].

## 3. Results

### 3.1. Characteristics of Study Sample

Among 622 men with PC included in the analysis, 133 (21%) were Black and 489 (79%) were White. Characteristics of men with PC are shown in Table 1. At diagnosis, Black men were more likely to have stage 4 and grade 3 compared to White men.

During follow-up (median 11.6 years, range 0–29.1 years), 350 men died; among them, 74 died from PC as the underlying cause. In accordance with a higher percentage of Blacks diagnosed at later stage in our study and a more aggressive course of disease [25], PC-specific mortality rate was slightly higher in Black men despite lower all-cause mortality (Table 1).

Frequency of alleles for four SNPs under study—*HSD3B1* rs1047303, *SRD5A2* rs523349, *SLCO2B1* rs1789693 and *SLCO2B1* rs12422149—are summarized in Table 2 for all men and by race. The frequency of major allele for rs12422149 was similar in Whites and Blacks, while between-race differences were observed for the other SNPs, especially for rs1789693 and rs1047303, which is consistent with known patterns [26].

### 3.2. Univariate Analysis of Cancer-Specific and All-Cause Survival

**Figure 1:**
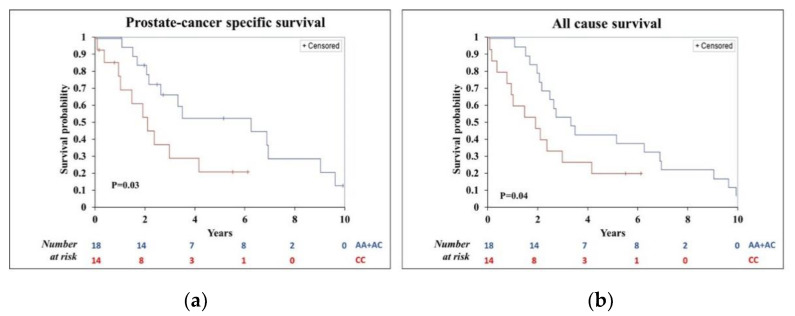
Prostate cancer (PC)-specific and all-cause survival for CC/CA vs. AA genotype in *HSD3B1* rs1047303 in men diagnosed with stage 4, metastatic PC. (**a**) PC-specific survival; (**b**) All-cause survival.

In univariate Kaplan-Mayer analysis, PC-specific and overall survival did not differ between carriers (CC/CA) and non-carriers (AA) of the minor allele C in *HSD3B1* rs1047303, overall, including in Whites (Figure S1). However, for men diagnosed with metastatic PC (stage 4), both PC-specific and all-cause survival were significantly worse for carriers of minor allele (C) (Figure 1a,b). For *SLCO2B1* rs1789693, PC-specific and all-cause survival appeared to differ between non-carriers (AA) and carriers (AT/TT) of the minor allele T, with statistically significant worse all-cause survival overall including in Whites (Figure S2). Neither PC-specific survival, nor all-cause survival differed by carrier status for the other two SNPs (Figures S3 and S4).

### 3.3. Multivariable Analysis of PC-Specific Mortality and All-Cause Mortality for Individual SNPs and a Priori Genetic Risk Score (GRS)

#### 3.3.1. PC-Specific Mortality Associated with Individual SNPs

The minor C allele of rs1047303 was not statistically significantly associated with PC-specific mortality overall or in White men (Table 3). However, there was an increased hazard ratio for 10-year mortality (per risk allele, HR = 2.54, *p* = 0.07, including in Whites HR = 2.72; *p* = 0.002, Table S1).

Importantly, the association was statistically significant among men diagnosed with metastatic (stage 4) PC: (HR = 4.89, 95% CI: 1.44–16.11, per risk allele, *p* = 0.01, not presented in tables).

For the A (vs. T) allele of *SLCO2B1* rs1789693, HR = 1.46 per risk allele (*p* = 0.10) including significant associations in Whites (HR = 1.70 per risk allele, *p* = 0.04, Table 3). Likewise, for the AA vs. AT/TT genotype, HR = 1.53 (*p* = 0.04) including in Whites (HR = 2.23 per risk allele, *p* = 0.006, Table S2). For *SLCO2B1* rs12422149, the G allele was not significantly associated with PC-specific mortality overall or in Whites for the whole follow-up. However, for the 10-year PC-specific mortality, HR = 2.85 per risk allele (*p* = 0.05), including a significant association in Whites HR = 3.54 (*p* = 0.04, Table S1). There were no associations between *SRD5A2* rs523349 and PC-specific mortality (Table 3).

#### 3.3.2. All-Cause Mortality Associated with Individual SNPs

Rs1047303 was not associated with all-cause mortality (Table S3); however, among men with stage 4 at diagnosis, the C allele was statistically significantly associated with a higher risk of all-cause mortality (HR = 4.74, per risk allele, *p* = 0.01, not presented in the tables). For A allele of *SLCO2B1* rs1789693, HR = 1.17 per risk allele (*p* = 0.10), including a marginally significant association in White men (HR = 1.23, *p* = 0.05). All-cause mortality was not associated with *SLCO2B1* rs12422149 or *SRD5A2* rs523349 (Table S3).

#### 3.3.3. GRS and PC-Specific and All-Cause Mortality

One unit increase in GRS was associated with a statistically significant increase in PC-specific mortality overall (by 26%) including in Whites (by 39%, Table 3). For men in the highest vs. lowest GRS tertile, the HRs were 1.73 (*P*_trend_ = 0.03) overall and 3.21 (*P*_trend_ = 0.01) in Whites. GRS was not associated with PC-specific mortality in Black men but the sample size was limited. In addition, we found an improvement in the model fit of the multivariable model including GRS vs. the model without GRS (*p* = 0.02). The magnitude of the improvement was similar but slightly greater than the improvement attained by adding age at PC diagnosis to the model adjusted for center, stage and grade at diagnosis.

Additionally, the association was stronger for the 10-year PC-specific mortality. For instance, in Whites, HR = 1.59 per one risk allele and HR = 5.29 (*P*_trend_ = 0.005) for the highest vs. lowest GRS tertile (Table S1). Further, the associations with PC-specific mortality were similar when we re-ran the analysis using the follow-up until 2012—the time of abiraterone adoption in the U.S. [1,24]. Finally, GRS was not statistically significantly associated with all-cause mortality (Table S3).

## 4. Discussion

This study demonstrates that a variant in *HSD3B1* (rs1047303C, also called 1245C), a gain-of-function allele driving androgen production, is associated with increased PC-specific and all-cause mortality in men with metastatic PC in a population-based (and not disease-selected) cohort. This is in line with the previous findings in clinical trials of PC patients receiving ADT [5,10,11,27]. This finding makes rs1047303 a very important variant because although several effective treatments exist for castration sensitive prostate cancer (CSPC), treatment resistance is nearly universal as is eventual death from the disease. Thus, this SNP may help to monitor disease progression, predict disease recurrence and therapeutic treatment efficacy.

Further, using a novel approach, we showed a statistically significant association of a priori genetic risk score (GRS) based on the sum of the four pre-selected androgen-regulating polymorphisms with greater PC-specific mortality. This result suggests, for the first time, the presence of a cumulative impact of these variants involved in androgen production, uptake or conversion (APUC) on PC-specific mortality independent of stage and grade at diagnosis. Stronger associations for PC-specific mortality (vs. all-cause mortality) suggests that these polymorphisms collectively contribute to the progression of PC.

Our finding for *HSD3B1* (1245C) is supported by a biological mechanism. Carriers of the 1245C allele, which encodes a more stable form of 3βHSD1, will have increased production of androgen precursors. In PC cells, androgen precursors are converted into DHT—the major driver of PC [3,28,29]. Thus, higher levels of the precursors among carriers of the 1245C would lead to increased DHT levels and enhance tumor growth, further contributing to the risk of metastatic progression and mortality.

The finding for *HSD3B1* (1245C) in metastatic PC also agrees with previous studies of PC patients receiving ADT. Hearn et al. found that men with hormone-sensitive PC who carry one or two C alleles had worse progression-free survival vs. those with AA genotype: HRs were 2.8 (95%CI: 1.1–6.7; *p* = 0.03) and 3.5 (95%CI: 1.3–9.5; *p* = 0.01), for metastases development and death, respectively [5]. This finding was replicated in subsequent U.S. [4,10] and Japanese [30] studies of PC patients treated with ADT. Recently, *HSD3B1* (1245C) was also shown to be associated with a higher overall mortality in low-volume metastatic cancer [11]. In our study, the association with *HSD3B1* (1245C) was found among those with metastatic PC. This may be explained by the fact that, compared to those with metastatic cancer, most individuals with localized PC survive longer after diagnosis and die not from PC, and other factors may have a stronger impact on their mortality than *HSD3B1* (1245C). It is also likely that 1245C predicts resistance to standard ADT and most (if not all) patients with metastatic disease are treated with ADT.

Despite convincing evidence for the effect of *HSD3B1* (1245C) on PC progression among patients treated with ADT, the data are less consistent for patients receiving more novel anti-androgen treatment—abiraterone—a selective CYP17A1 inhibitor that reduces extra-gonadal synthesis of androgen [30,31,32,33]. To ensure that treatment heterogeneity did not influence our findings, we restricted the follow-up to 2012, the time of the abiraterone adoption in the U.S., and the associations with GRS mirrored those for the full follow-up until 2019. Thus, abiraterone adoption did not influence our findings, especially, because it is unlikely that many patients were treated with abiraterone in this study given the timeline of PC diagnosis in 1987–2015.

Further, we detected a higher risk of both PC-specific and all-cause mortality associated with having A allele in *SLCO2B1* rs1789693 in the whole cohort and among White men. Our findings are consistent with some [14], but not all studies of patients on ADT [12,13]. Additionally, in our study, more active *SLCO2B1* rs12422149 G allele was associated with higher 10-year PC-specific mortality, which agrees with studies of PC patients receiving ADT [13,14,34]. The observed association is also supported by in vitro studies that found higher transporting activity of the DHEA sulfate for G vs. A allele, that led to enhanced intracellular androgen ability resulting in androgen receptor activation and increased cell proliferation [13].

We did not find associations for *SRD5A2* rs523349, while two previous studies reported contradictory findings with this polymorphism in patients on ADT [6,15]. Multiple explanations may account for such discrepant findings including different ethnicities, various outcomes, different treatments, and the lack of adjustment in several studies, contributing to biased estimates. Finally, the biological mechanisms underlying the interplay between uptake, transport and conversion of androgen-regulating genes is incompletely characterized. Thus, larger studies coupled with examining biological mechanisms are needed to clarify the cumulative role of these polymorphisms in PC progression.

Allele frequencies for three SNPs under study differed by race. Black men, for example, tended to harbor the risk 1245C allele in *HSD3B1* (Table 2). This confirms that Black men are more likely to have androgen receptor mutations than White men [35], which may be driving hormonal responsiveness and aggressive disease in Black vs. White men. It is possible that the effect of the APUC variants differs by race, although a limited sample in Blacks precluded us from getting reliable estimates. Further studies of between-race relationships are warranted.

A strength of this study includes pathologically confirmed PC cases that were thoroughly followed from 1987–2019, validated information about causes of death and detailed information about clinical and demographic characteristics. Limitations include lack of detailed information on treatment, and low power in subgroups especially in Blacks. Further, we cannot exclude the possibility of false positive associations, so our findings need to be validated in the future large studies. This being said, an important strength and novelty of our population-based cohort study is that it included a different, more diverse population, wider ranges of comorbidities and longer follow compared to therapeutic trials, which increases generalizability to the broader population of individuals with PC.

## 5. Conclusions

This study confirms the effect of the critical androgen-regulating variant—*HSD3B1* 1245C—in the PC prognosis in the population-based cohort of White and Black men and suggests a combined role of the androgen pathways (production, uptake or conversion) in PC-specific mortality, which merits consideration. As hypothesized, these polymorphisms may contribute to variation in a chain of hormonal events that may drive an androgen rich tumor milieu promoting PC progression.

## Figures and Tables

**Table 1:** Characteristics of men diagnosed with PC stratified by race, ARIC 1987–1989.

Characteristics	All Men	White Men	Black Men	*p*-Value ^1^
Number of men	622	489	133	
Mean age at diagnosis (SD), y	69.7 (6.3)	69.9 (6.3)	69.8 (6.3)	<0.12 ^2^
Median (range)	70 (53–86)	70 (53–86)	68 (54–84)	
Stage at diagnosis ^3^ (%)				0.14
1 and 2 combined 3 4	82.0 12.2 5.8	82.8 12.3 4.9	79.2 11.2 9.6	
Grade at diagnosis ^3^ (%)				0.0005
1 2 3 Unknown	20.6 46.1 27.7 5.6	21.9 48.9 24.1 5.1	15.8 36.1 40.6 7.5	
First course of treatment ^4^				
Surgery (yes, %) Radiation (yes, %) Hormonal therapy (yes, %)	51.3 17.6 25.4	51.3 20.0 25.0	51.1 16.9 26.7	0.98 0.45 0.75
Co-morbidities at baseline				
Diabetes (yes, %) Cardiovascular disease (yes, %)	8.1 6.4	8.1 7.0	8.0 4.0	0.96 0.14
Co-morbidities before diagnosis				
Diabetes (yes, %) Cardiovascular disease (yes, %)	14.0 12.1	13.7 12.5	15.3 10.4	0.65 0.15
Number of deaths	350	289	61	
Incidence rate of death from any cause (per 1000 men)	47.9	49.7	41.1	
Incidence rate of PC-specific death (per 1000 men)	10.2	10.0	11.0	
Median follow-up time (range, y)	11.6 (0–29.1)	11.0 (0.11–29.1)	10.5 (0–27.7)	

^1^*p*-value was calculated using chi-square test unless stated otherwise. ^2^*p*-value was calculated using *t*-test. ^3^ Information about stage and grade at diagnosis were obtained from cancer registries: TNM stage (categorical variable 1 + 2 combined, 3 and 4) and SEER grade at diagnosis (categories 1–3), SEER grade includes the following categories: 1 (corresponds to Gleason sum ≤ 3), 2 (Gleason sum of 4–6), and 3 (Gleason grade ≥ 7). All the categories were determined a priori.

**Table 2:** Single nucleotide polymorphisms (SNPs) in the androgen-regulating genes, ARIC.

SNP	Gene (Chromosome) Function	Major Allele	Minor Allele	Frequency of Major Allele			Risk Allele ^1^
				All	White Men	Black Men	
rs1047303 (1245C) Missense C–gain of function	*HSD3B1* (1) Androgen synthesis and production	A	C	0.72	0.68	0.88	C
rs1789693 Intron	*SLCO2B1*(11) Androgen transport and uptake	A	T	0.59	0.66	0.34	A ^2^
rs12422149 Missense	*SLCO2B1* (11) Androgen transport and uptake	G	A	0.90	0.90	0.90	G
rs523349 Missense	*SRD5A2* (2) Androgen conversion	C	G	0.73	0.70	0.76	G ^2^

^1^ Risk allele was associated with androgen activation or with the metabolism of hormone therapy for PC [12]. ^2^ The associations with PC progression in published studies were inconsistent for rs1789693 [13,14] and rs523349 [6,15].

**Table 3:** Associations between SNPs located in the androgen-regulating genes and PC-specific mortality in Black and White men with PC; ARIC 1987–2019.

Polymorphisms	Hazard Ratio and 95% Confidence Intervals for PC-Specific Mortality ^1^		
	All	White Men	Black Men
No. of men with PC	622	489	133
No. of PC-specific deaths	74	58	16
Person-years (per 1000 person-years)	7273	5815	1459
SNP (risk allele) ^2^			
rs1047303 (C)	1.40 (0.96–2.06) *p* = 0.08	1.39 (0.93–2.08) *p* = 0.11	1.89 (0.55–6.48) *p* = 0.76
rs1789693 (A)	1.46 (0.93–2.27) *p* = 0.10	1.70 (1.01–2.85) *p* = 0.04	0.45 (0.15–1.31) *p* = 0.14
rs12422149 (G)	1.22 (0.69–2.18) *p* = 0.49	1.15 (0.62–2.12) *p* = 0.66	1.98 (0.28–13.85) *p* = 0.50
rs523349 (G)	1.05 (0.73–1.51) *p* = 0.76	1.26 (0.83–1.91) *p* = 0.27	0.95 (0.43–2.11) *p* = 0.88
Genetic Risk Score, GRS ^3^			
Tertiles			
0.12–3.76 3.77–4.82 4.83–7.68	1 (Reference) 1.39 (0.76–2.55) 1.73 (0.95–3.16)	1 (Reference) 2.71 (1.15–6.40) 3.21 (1.46–7.07)	1 (Reference) 0.80 (0.20–3.16) 0.47 (0.06–3.82)
Continuous (per risk allele)	1.26 (1.02–1.56)	1.39 (1.08–1.77)	0.85 (0.51–1.42)
P_-trend_^4^	0.03	0.01	0.53

^1^ The SNPs (risk allele) are presented using an additive model. ^2^ Hazard ratios are presented per one risk allele. Adjusted for age at diagnosis, center, stage and grade at diagnosis, and race, if appropriate. ^3^ Genetic risk score (GRS) was created as the sum of four SNPs (using additive model). The GRS was examined as continuous and categorized into tertiles. Tertiles were calculated using the whole analytical sample that combined White and Black men. ^4^ P_-trend_ was calculated by putting categories for GRS as an ordinal variable into the model.

## Data Availability

The data set supporting the results of this article is available in the dbGaP repository, phs000280.v5.p1 (https://www.ncbi.nlm.nih.gov/projects/gap/cgi-bin/study.cgi?study_id=phs000280.v5.p1).

## Supplementary Materials

The following are available online at https://www.mdpi.com/article/10.3390/cancers13081958/s1, Figure S1: Prostate cancer-specific and all-cause survival overall and in White men with prostate cancer for HSD3B1 rs1047303, Figure S2: Prostate cancer-specific and all-cause survival overall and in White men with prostate cancer for SLCO2B1 rs1789693, Figure S3: Prostate cancer-specific and all-cause survival overall and in White men with prostate cancer for SLCO2B1 rs12422149, Figure S4: Prostate cancer-specific and all-cause survival overall and in White men with prostate cancer for SRD5A2, Table S1: Associations between SNPs (additive model) in the androgen-regulating genes and 10-year prostate cancer (PC)-specific mortality; Table S2: Associations between SNPs (dominant model for minor allele) in the androgen-regulating genes and prostate cancer (PC)-specific mortality in men with prostate cancer; Table S3: Associations between SNPs (additive model) in the androgen-regulating genes and all-cause mortality in men with prostate cancer.
