# Supplementary material for: Prostate Cancer Mortality Associated with Aggregate Polymorphisms in Androgen-Regulating Genes: The Atherosclerosis Risk in the Communities (ARIC) Study
Source: Cancers (Basel). 2021 Apr 19;13(8):1958. doi: 10.3390/cancers13081958 (PMC8072683; doi:10.3390/cancers13081958)
Supplement: Supplementary file 1 [file cancers-13-01958-s001.zip › cancers-1181463-supplementary.pdf]

cancers

Article

Prostate Cancer Mortality Associated with Aggregate Polymorphisms in Androgen-Regulating Genes: the Atherosclerosis Risk in the Communities (ARIC) Study

Anna E. Prizment [1,2,*], Sean McSweeney [3], Nathan Pankratz [4], Corinne E. Joshu [5,6], Justin H. Hwang [1], Elizabeth A. Platz [5,7] and Charles J. Ryan [1,2]

1 Division of Hematology, Oncology and Transplantation, University of Minnesota Medical School, Minneapolis, MN 55455, USA; jhwang@umn.edu (J.H.H.); ryanc@umn.edu (C.J.R.)
2 University of Minnesota Masonic Cancer Center, Minneapolis, MN 55455, USA
3 University of Minnesota Medical School, Minneapolis, MN 55455, USA; mcswe027@umn.edu
4 Department of Laboratory Medicine and Pathology, University of Minnesota, Minneapolis, MN 55455, USA; pankr018@umn.edu
5 Department of Epidemiology, Johns Hopkins Bloomberg School of Public Health, Baltimore, MD 21205, USA; cjoshu1@jhu.edu (C.E.J.); eplatz1@jhu.edu (E.A.P.)
6 Sidney Kimmel Comprehensive Cancer Center at Johns Hopkins, Baltimore, MD 21287, USA
7 Department of Urology and the James Buchanan Brady Urological Institute, Johns Hopkins University School of Medicine, Baltimore, MD 21287, USA
* Correspondence: prizm001@umn.edu

Citation: Prizment, A.E.; McSweeney, S.; Pankratz, N.; Joshu, C.E.; Hwang, J.H.; Platz, E.A.; Ryan, C.J. Prostate Cancer Mortality Associated with Aggregate Polymorphisms in Androgen-Regulating Genes: the Atherosclerosis Risk in the Communities (ARIC) Study. *Cancers* **2021**, *13*, 1958. https://doi.org/10.3390/cancers13081958

Academic Editor: Pierre Jean Lamy, Christophe Hennequin, Mathieu Roumiguie, Xavier Rebillard and Carlos S. Moreno

Received: 29 March 2021
Accepted: 15 April 2021
Published: 19 April 2021

Publisher's Note: MDPI stays neutral with regard to jurisdictional claims in published maps and institutional affiliations.

Copyright: © 2021 by the authors. Submitted for possible open access publication under the terms and conditions of the Creative Commons Attribution (CC BY) license (http://creativecommons.org/licenses/by/4.0/).

Supplementary

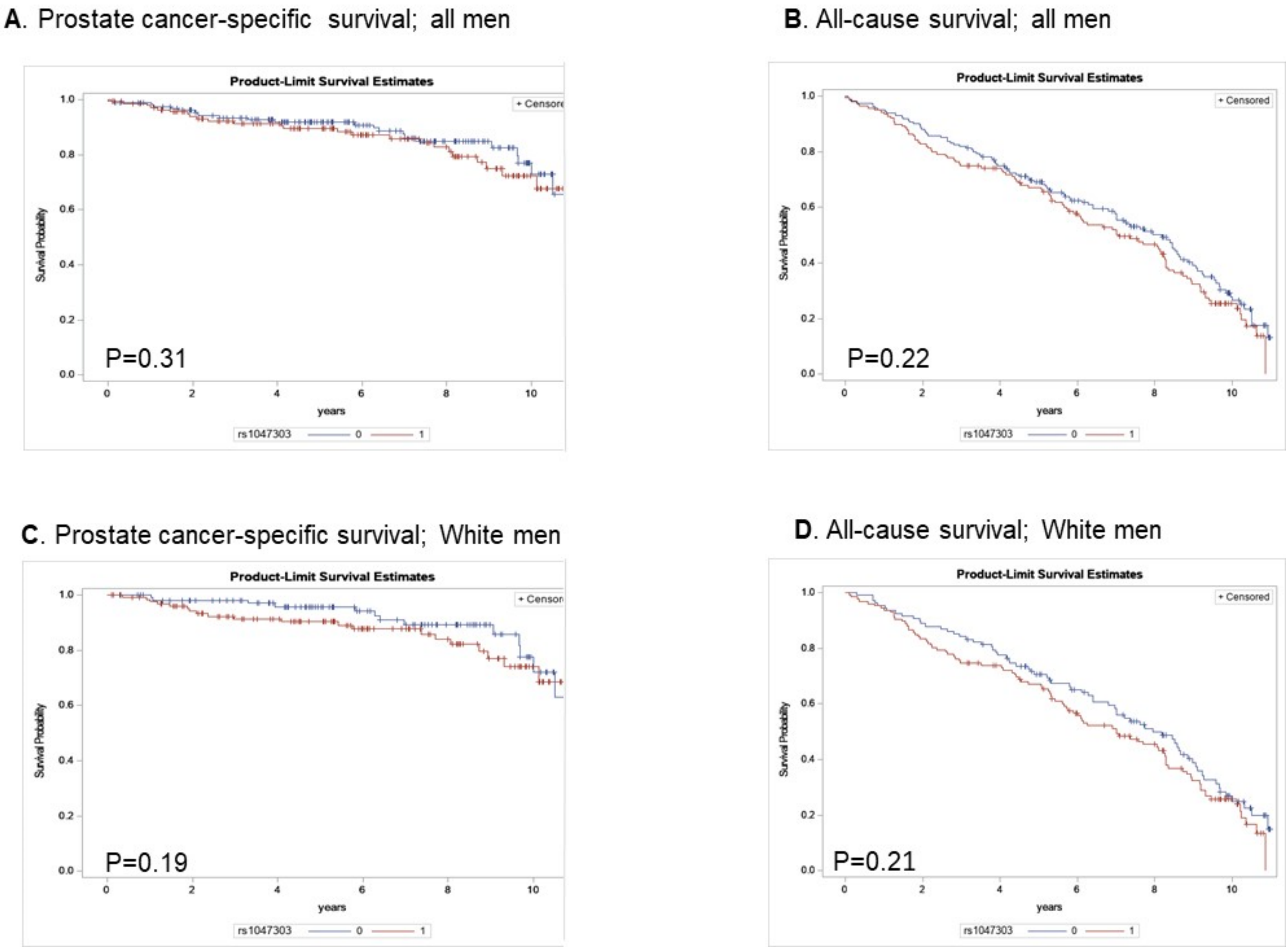

Figure S1. Cumulative prostate cancer-specific and all-cause survival of white men with prostate cancer for *HSD3B1* rs1047303. CC/CA genotype (1, risk) vs. AA genotype (0).

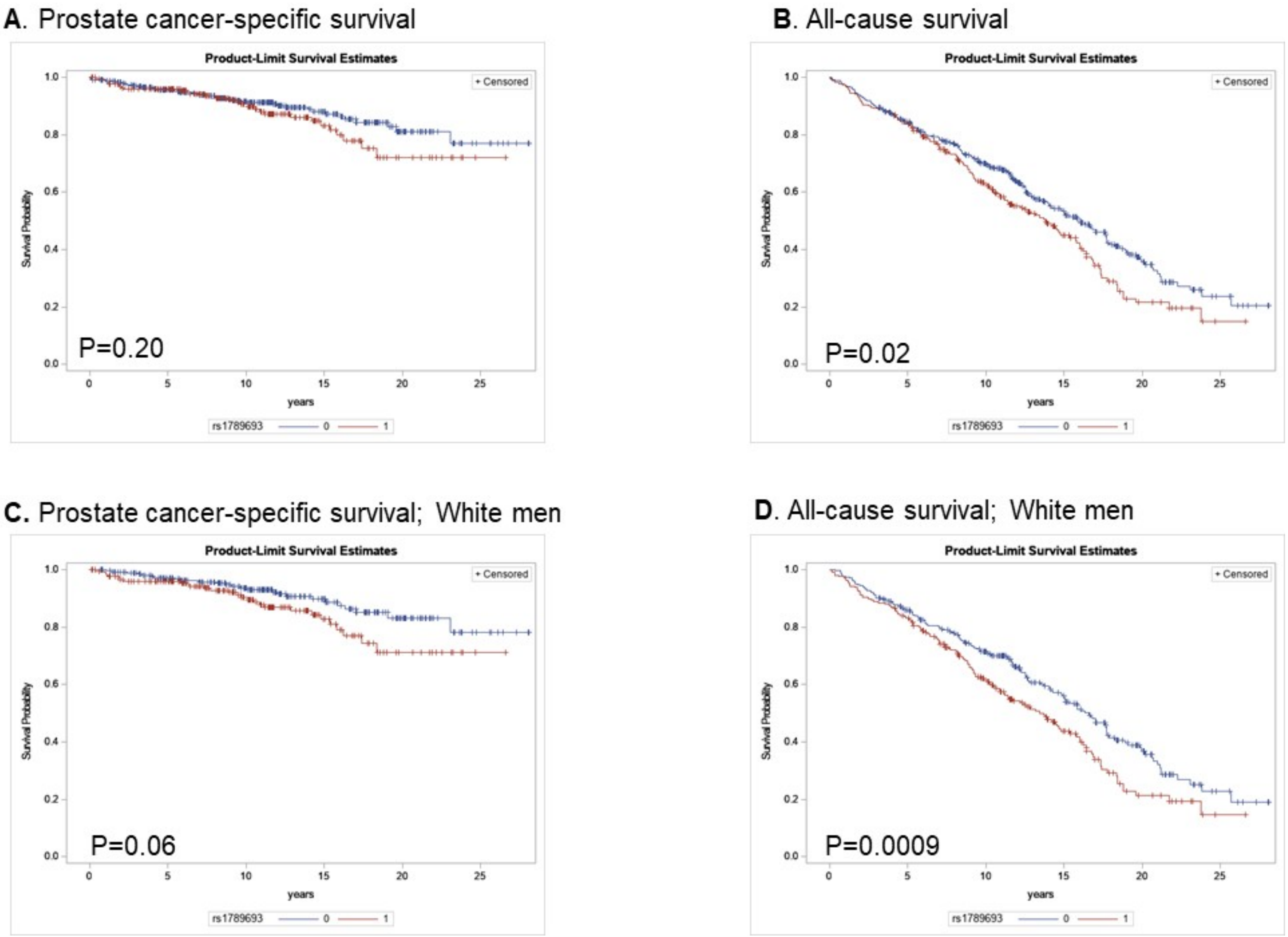

Figure S2. Cumulative prostate cancer-specific and all-cause survival of White men with prostate cancer for *SLCO2B1* rs1789693. AA genotype (1, risk) vs. AT/TT genotype (0).

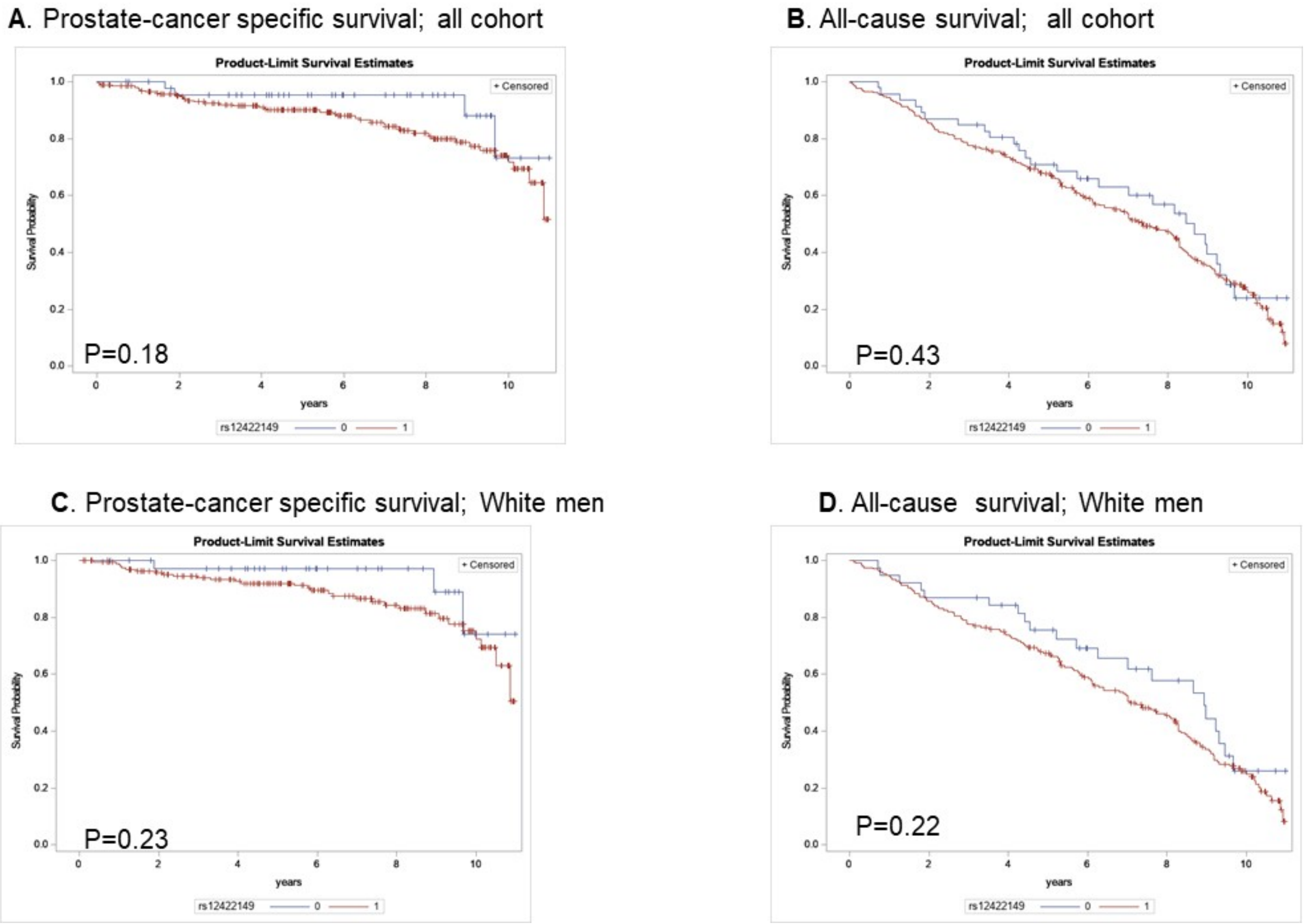

Figure S3. Cumulative prostate cancer-specific and all-cause survival overall (A, B) and in White men (C, D) with prostate cancer for *SLCO2B1* rs12422149, GG (1-risk) vs. AA/GA (0). P-value was computed using log-rank test.

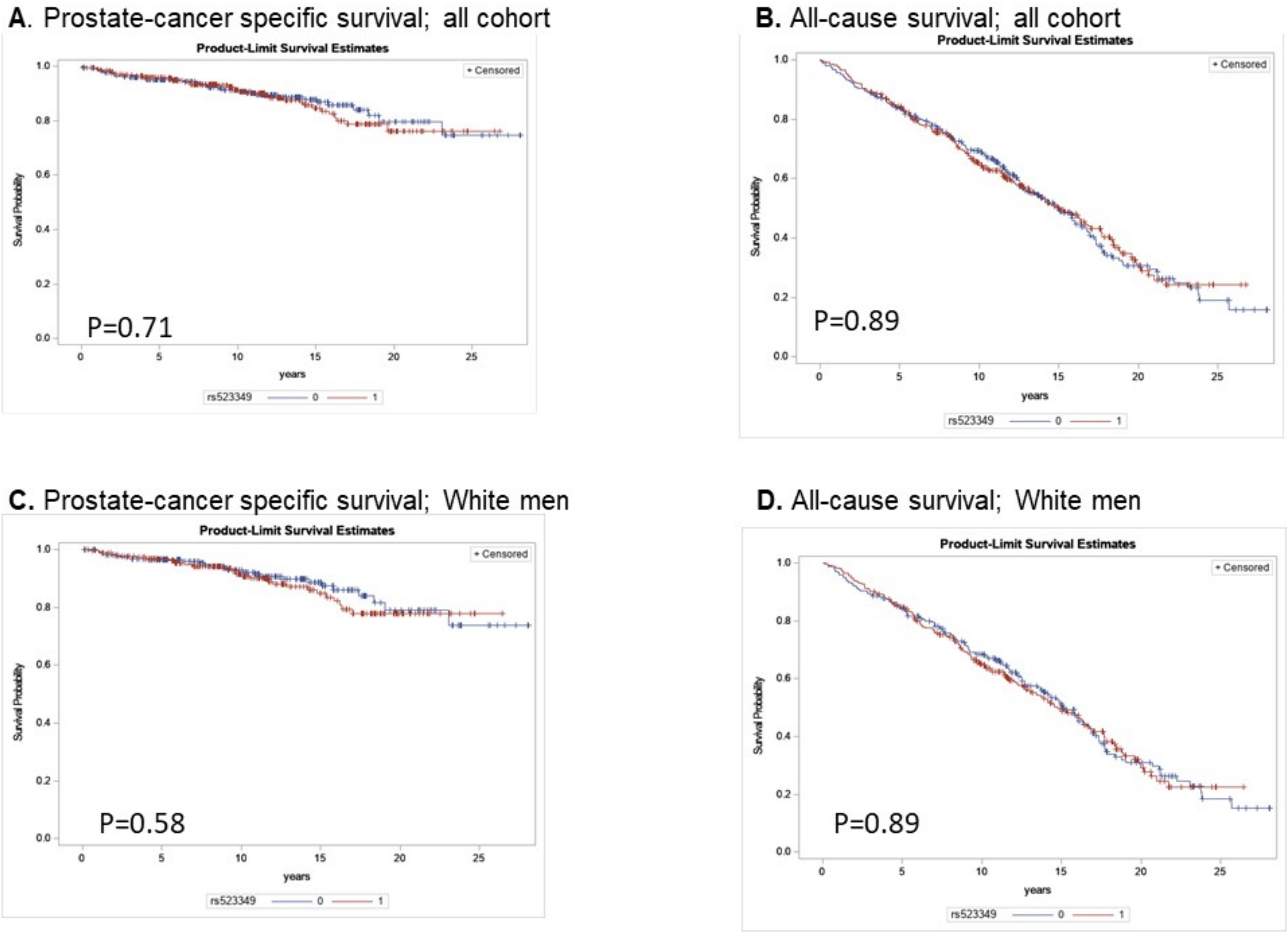

Figure S4. Cumulative prostate cancer-specific and all-cause survival overall (A, B) and in White men (C, D) with prostate cancer for *SRD5A2* rs523349 GG/GC (1-risk) vs. CC (0). P-value was computed using log-rank test.

Table S1. Associations between SNPs (additive model) in the androgen-regulating genes and 10-year prostate cancer (PC)-specific mortality.

Polymorphisms	Hazard ratio (95% confidence intervals) for PC-specific mortality[2]		
	All	White men	Black men
Number of men with PC	622	489	133
Number of PC-specific deaths	47	33	14
Person-years (per 1000 person-years)	1286	1086	300
SNPs (risk allele) [1]			
rs1047303 (C)	2.54 (1.43-4.52); P =0.07	2.72 (1.42-5.18); P =0.002	1.20 (0.17-8.70); P=0.86
rs1789693 (A)	1.03 (0.58-1.82); P=0.93	1.46 (0.71-3.01); P=0.30	0.31 (0.08-1.12); P=0.07
rs12422149 (G)	2.85 (1.03-7.89); P=0.05	3.54 (1.09-11.44); P=0.04	2.46 (0.35-17.1); P=0.36
rs523349 (G)	0.69 (0.41-1.15); P=0.15	0.80 (0.44-1.46); P=0.46	0.46 (0.16-1.35); P=0.89
Genetic Risk Score, GRS[3]			
Tertiles			
0.12 – 3.76	1 (Reference)	1 (Reference)	1 (Reference)
3.77 – 4.82	1.48 (0.71-3.10)	4.58 (1.47-13.30)	0.31 (0.06-1.62)
4.83 – 7.68	1.83 (0.83-4.03)	5.29 (1.79-15.59)	0.24 (0.03-2.56)
Continuous (per risk allele)	1.24 (0.95-1.63)	1.59 (1.15-2.19)	0.72 (0.43-1.22)
$P_{for\ trend}$ [4]	0.12	0.005	0.22

[1] The SNPs (risk allele) are presented using an additive model. [2] HR are presented per one allele increase in the risk allele. Adjusted for age at diagnosis, center, stage and grade at diagnosis, and race, if appropriate. [3] Genetic risk score (GRS) was created as the sum of four SNPs (using additive model). The GRS was examined as continuous and categorized into tertiles. Tertiles were calculated using the whole analytical sample that combined White and Black men. [4] $P_{for\ trend}$ was calculated by putting categories for GRS as an ordinal variable into the model.

Table S2. Associations between SNPs (dominant model for minor allele)[1] in the androgen-regulating genes and prostate cancer (PC)-specific mortality in men with PC.

Polymorphisms	Hazard ratio (95% confidence interval) for PC-specific mortality[2]		
	All men	White men	Black men
Number of men with PC	622	489	133
Number of PC-specific deaths	74	58	16
Person-years (per 1000)	7273	5815	1459
rs1047303 (1245C); CC/CA vs. AA	1.43 (0.85-2.41); P = 0.17	1.47 (0.84-2.56); P = 0.17	0.80 (0.16- 4.20); P = 0.80
rs1789693; AA vs. AT/TT	1.53 (1.03-2.35); P = 0.04	2.23 (1.27-3.91); P = 0.006	0.57 (0.23-1.45); P = 0.24
rs12422149; GG vs. AA/GA	1.21 (0.63-2.49); P = 0.57	1.14 (0.56-2.31); P = 0.72	2.25 (0.28-17.88); P = 0.44
rs523349; GG/GC vs. CC	1.15 (0.72-1.85); P = 0.57	1.43 (0.82-2.50); P = 0.21	0.95 (0.43-2.12); P = 0.90

[1]The SNPs were modeled as two groups: homozygous genotype of major allele versus minor homozygous + heterozygous genotypes combined (dominant model for minor allele). [2] HR are adjusted for age at diagnosis, center, stage and grade at diagnosis.

Table 3. Associations between SNPs (additive model) and genetic risk score in the androgen-regulating genes and all-cause mortality in men with prostate cancer.

**Polymorphisms**	**Hazard ratio (HR) and 95% confidence intervals (CI)[2] for all-cause mortality**		
	**All men**	**White men**	**Black men**
Number of men with prostate cancer	622	489	133
Number of all-cause deaths	350	289	61
Person-years (per 1000)	7273	5815	1459
SNP (risk allele) [1]			
rs1047303 (C)	1.03 (0.80-1.32); P=0.83	1.06 (0.90-1.25); P=0.50	1.06 (0.90-1.25); P=0.50
rs1789693 (A)	1.17 (0.97-1.42); P=0.10	1.23 (1.00-1.52); P=0.05	0.75 (0.47-1.18); P=0.22
rs12422149 (G)	1.03 (0.80-1.32); P=0.83	1.03 (0.79-1.35); P=0.82	0.92 (0.46-1.86); P=0.82
rs523349 (G)	0.97 (0.82-1.15); P=0.72	1.06 (0.88-1.28); P=0.56	0.67 (0.44-1.02); P=0.06
Genetic Risk Score[3]			
Tertiles			
0.12 – 3.76	1 (Reference)	1 (Reference)	1 (Reference)
3.77 – 4.82	1.15 (0.88-1.52)	1.22 (0.89-1.70)	1.03 (0.54-1.99)
4.83 – 7.68	1.16 (0.87-1.53)	1.26 (0.92-1.72)	0.43 (0.13-1.42)
Continuous (per risk allele)	1.05 (0.96-1.15)	1.10 (0.98-1.21)	0.81 (0.63-1.03)
$P_{for\ trend}$ [4]	0.22	0.09	0.09

[1] The SNPs (risk allele) are presented using an additive model. [2] HR are presented per one allele (additive model). Adjusted for age at diagnosis, center, stage and grade at diagnosis, and year at diagnosis. [3] Genetic risk score was created as the sum of four SNPs (using additive model). The GRS was examined as continuous and categorized into tertiles. Tertiles were calculated using the whole analytical sample that combined White and Black men. [4] $P_{for\ trend}$ was calculated by putting tertiles of genetic risk score as an ordinal variable into the model.
